# Correction: The *MIR157*–SPL15 module regulates flowering and inflorescence development in *Arabidopsis thaliana* under short days and in *Arabis alpina*

**DOI:** 10.1371/journal.pgen.1011978

**Published:** 2025-12-15

**Authors:** The PLOS One Staff

## Notice of Republication

This article was republished on November 26, 2025, to correct an error in Fig 6 that occurred during the production process. In the originally published version, panel 6E contained corrupted images. The publisher apologizes for the errors. Please download this article again to view the correct version. The originally published, uncorrected article and the republished, corrected articles are provided here for reference.

## Supporting information

S1 File Originally published, uncorrected article. (PDF)

S2 File Republished, corrected article. (PDF)
