# Supplementary figures and images for: The MIR157–SPL15 module regulates flowering and inflorescence development in Arabidopsis thaliana under short days and in Arabis alpina
Source: PLoS Genet. 2025 Sep 2;21(9):e1011799. doi: 10.1371/journal.pgen.1011799 (PMC12404414; doi:10.1371/journal.pgen.1011799)

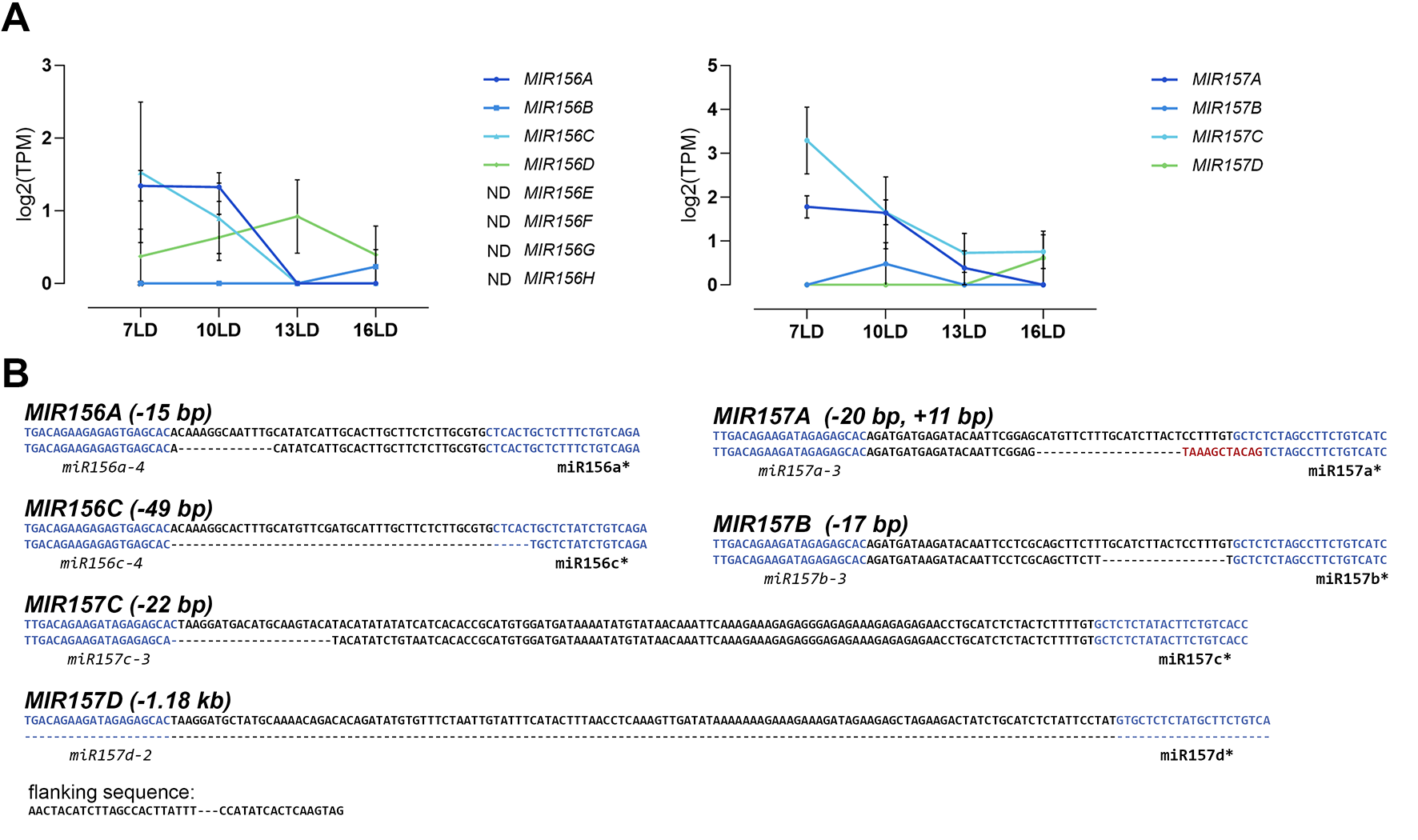

Supplement: S1 Fig — TPM denotes transcripts per kilobase of exon per million mapped fragments. Data retrieved from Cerise et al. 2023. (B) Sequence of the Arabidopsis CRISPR-Cas9 alleles generated in this study. All deletions affect the conserved hairpin sequence that is required for the correct biogenesis of miR156 and miR157. For each isoform, the wild-type reference sequence is shown above and the mutant sequence is shown below. The miRNA and miRNA* sequences are highlighted in blue. Red letters indicate non-template insertions. (TIFF) [file pgen.1011799.s004.tiff]

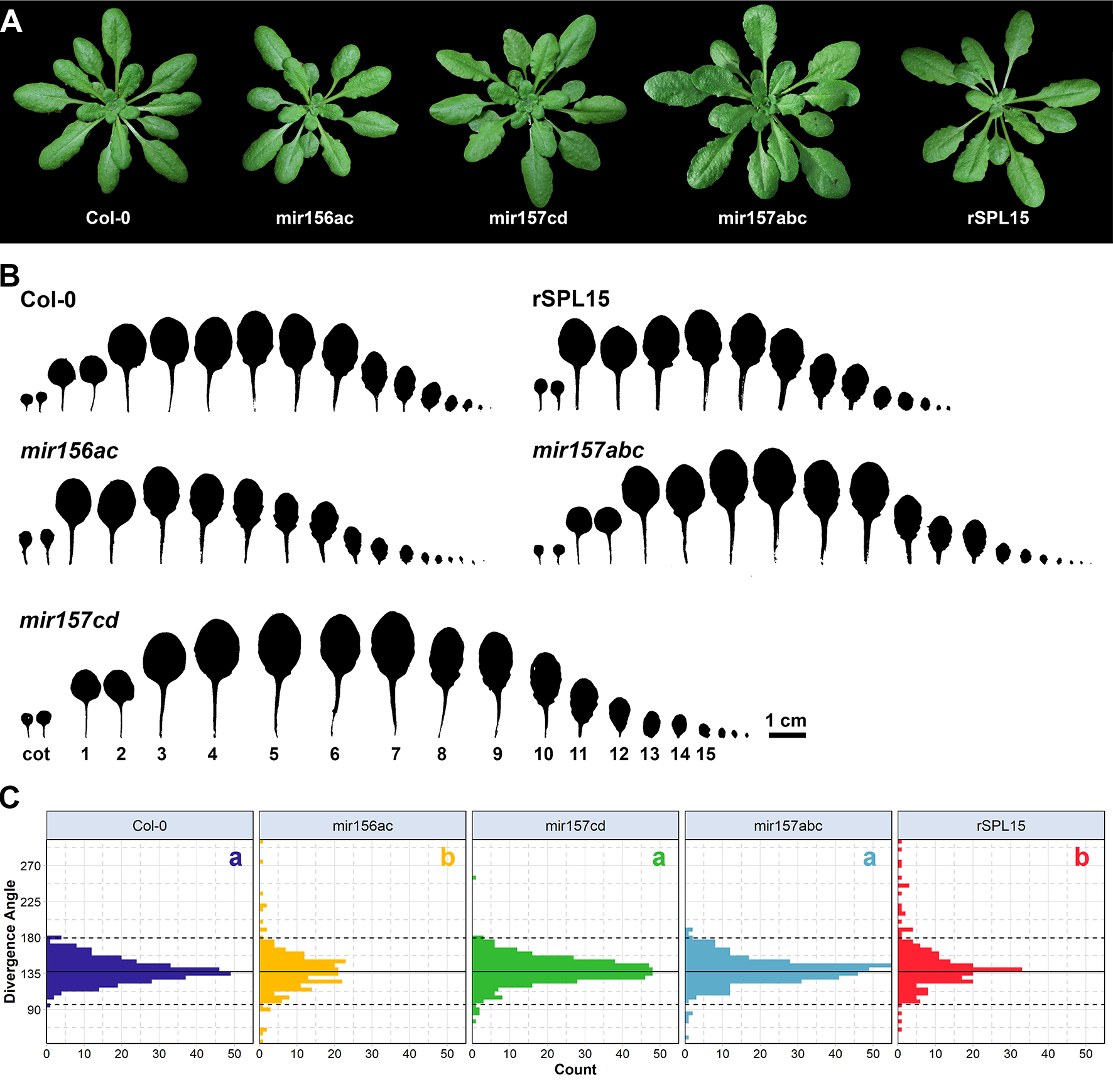

Supplement: S2 Fig — (A) Rosette phenotype of Col-0, mir156a-4 mir156c-4, mir157c-3 mir157d-2, mir157a-3 mir157b-3 mir157c-3 and rSPL15 plants grown for four weeks in short days (4wSD). (B) Morphology of fully expanded rosette leaves of wild-type plants and mutants for genes encoding miR156, miR157, and rSPL15 at 4wSD. (C) Distribution of the divergence angle between successive rosette leaves in 5wSD plants. The horizontal line indicates the ideal angle of 137.5° and dashed lines show the minimum and maximum angles measured for wild type. Letters indicate statistically significant differences in variation as calculated by Levene’s and Tukey’s post-hoc HSD tests. (TIFF) [file pgen.1011799.s005.tiff]

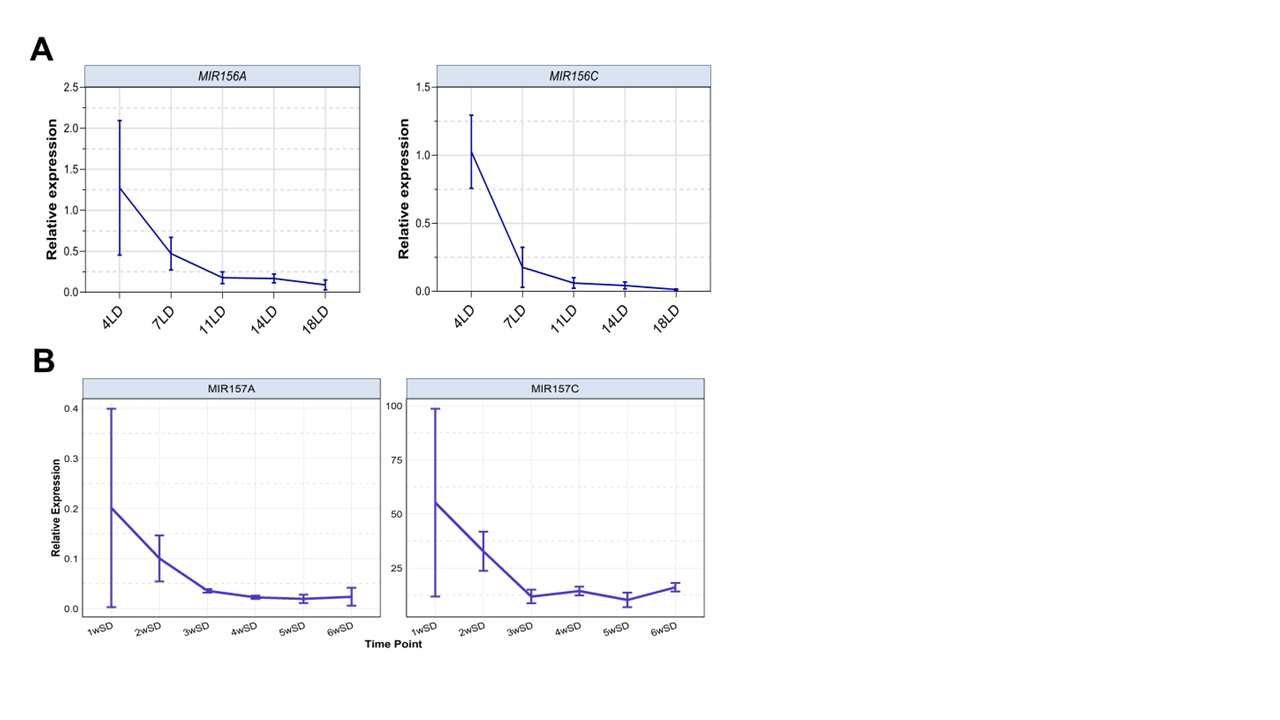

Supplement: S3 Fig — (B) Expression levels of endogenous MIR157A and MIR157C genes in apices of Col-0 plants grown in short days (SD). (TIFF) [file pgen.1011799.s006.tiff]

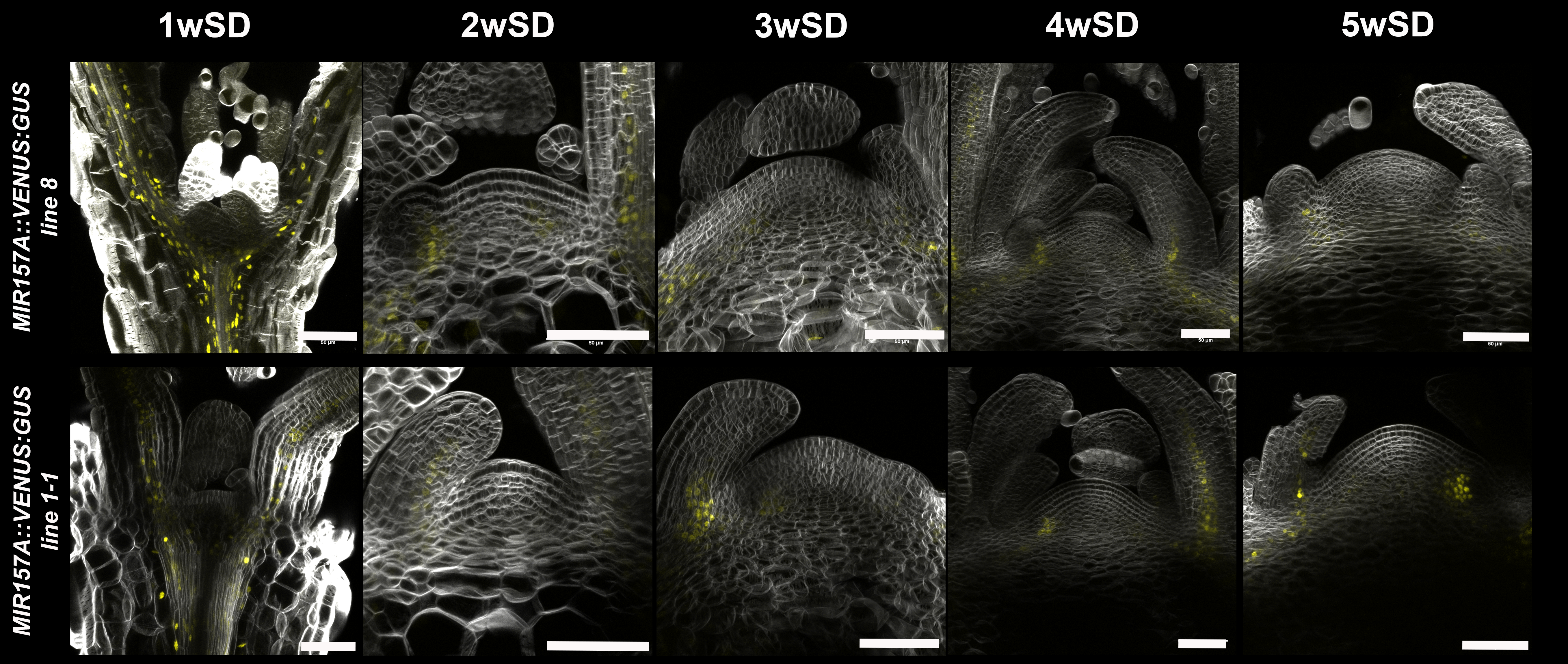

Supplement: S6 Fig — Confocal images showing the expression of MIR157A::VENUS:GUS in short days (SD). Fluorescence from the Venus protein is artificially coloured in yellow, and the fluorescence from the Renaissance dye is artificially coloured in grey. Scale bar = 50 µM. CL, Cauline leaf. (TIFF) [file pgen.1011799.s009.tiff]

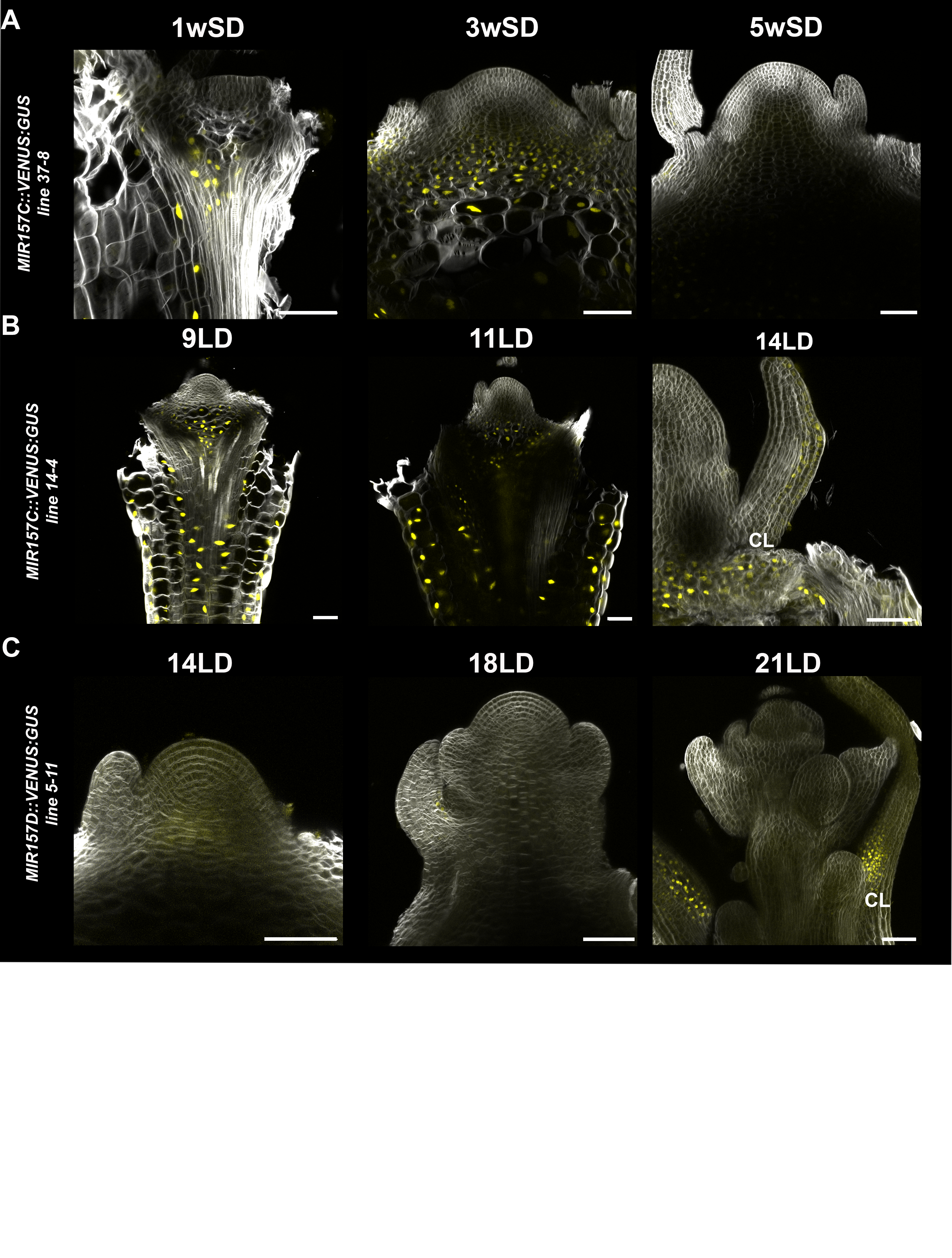

Supplement: S7 Fig — Confocal images showing the expression MIR157C::VENUS:GUS in (A) short days (SD), (B) long days (LD) and (C) MIR157D::VENUS:GUS in long days (LD). Fluorescence from the Venus protein is artificially coloured in yellow, and the fluorescence from the Renaissance dye is artificially coloured in grey. CL, Cauline leaf. Scale bar = 50 µM. (TIFF) [file pgen.1011799.s010.tiff]

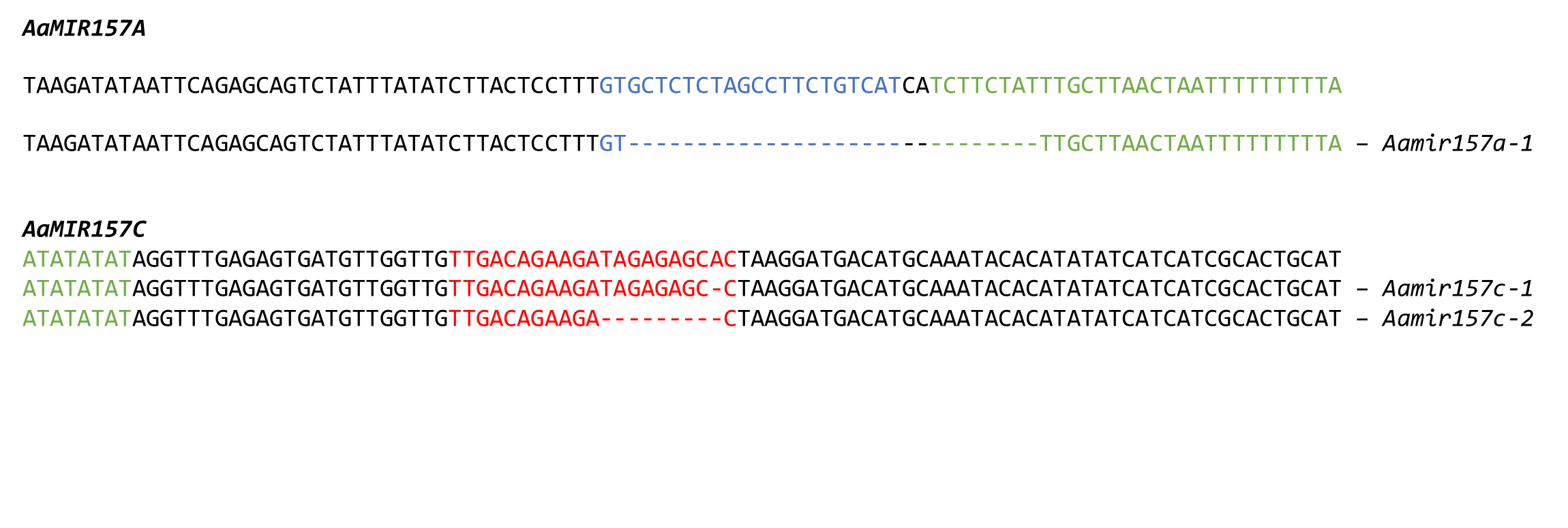

Supplement: S8 Fig — All deletions affect the conserved hairpin sequence that is required for the correct biogenesis of AamiR157. For each isoform, the wild-type reference sequence is shown above and the mutant sequence is shown below. The miRNA and miRNA* sequences are highlighted in red (AamiR157c) and blue (AamiR157a). Black letters indicate miRNA precursor and green letters indicate the genomic region. (TIFF) [file pgen.1011799.s011.tiff]
