# Supplementary material for: The MIR157–SPL15 module regulates flowering and inflorescence development in Arabidopsis thaliana under short days and in Arabis alpina
Source: PLoS Genet. 2025 Sep 2;21(9):e1011799. doi: 10.1371/journal.pgen.1011799 (PMC12404414; doi:10.1371/journal.pgen.1011799)
Supplement: Attachment — Submitted filename: Roggen Lloret Miotto et al Response to reviewers.docx [file pgen.1011799.s013.docx]

**Response to reviewers**

**We would like to thank the editor and reviewers for considering our manuscript, and for their valuable comments on the text and data. We respond to each of their comments below. Our responses are in bold and the reviewers’ and editor’s comments appear verbatim.**

**Editor**

Thank you for submitting your manuscript to PLOS Genetics. After careful consideration, we feel that it has merit but does not fully meet PLOS Genetics's publication criteria as it currently stands. Therefore, we invite you to submit a revised version of the manuscript that addresses the points raised during the review process.

Please submit your revised manuscript within 60 days (June 8). If you will need more time than this to complete your revisions, please reply to this message or contact the journal office at plosgenetics@plos.org. When you're ready to submit your revision, log on to <https://www.editorialmanager.com/pgenetics/> and select the 'Submissions Needing Revision' folder to locate your manuscript file.

Please include the following items when submitting your revised manuscript:

* A rebuttal letter that responds to each point raised by the editor and reviewer(s). You should upload this letter as a separate file labeled 'Response to Reviewers'. This file does not need to include responses to formatting updates and technical items listed in the 'Journal Requirements' section below.
* A marked-up copy of your manuscript that highlights changes made to the original version. You should upload this as a separate file labeled 'Revised Manuscript with Track Changes'.
* An unmarked version of your revised paper without tracked changes. You should upload this as a separate file labeled 'Manuscript'.

If you would like to make changes to your financial disclosure, competing interests statement, or data availability statement, please make these updates within the submission form at the time of resubmission. Guidelines for resubmitting your figure files are available below the reviewer comments at the end of this letter.

We look forward to receiving your revised manuscript.

**We appreciate the editor’s invitation to submit a revised version that addresses the points raised by the three reviewers. We have provided a point-by-point response to their comments below. We have uploaded a marked-up copy and a clean copy of the revised manuscript, as requested.**

**Reviewer #1**

In the manuscript ‘The MIR157–SPL15 module regulates flowering and inflorescence development in Arabidopsis thaliana under short days and in Arabis alpina, Roggen et al. present a detailed study on the contributions of miR156 and miR157 to different flowering-related phenotypes. While the role of miR156 in the regulation of the reproductive transition has been very well investigated, it was not yet clear to what extent MIR157 genes contribute to flowering-related processes. Roggen et al. show using small RNA-seq that RISC-loaded miR157 is not rapidly declining a few weeks after germination, but remains moderately present also later during development, in line with the expression dynamics of MIR156A/C and MIR157 C/D. Interestingly, they show that this role of MIR157C is conserved in the perennial Arabis alpina, where mutation of MIR157C results in early flowering. Using genetic approaches, Roggen et al. show that the activity of miR157 depends on SPL15 (like that of miR156) and that the miR157-SPL15 module plays a role when the FT pathway is not induced (i.e. under short days). The results are clearly presented and the manuscript has been written very well.
We (senior and junior reviewers) only have a few minor comments/suggestions:

**We thank the reviewer for the positive comments on the data and the manuscript, and appreciate the suggestions for improvements to the manuscript.**

- It is not entirely clear why different mutant combinations have been generated. It would be good to add a graph in the Suppl. data with the expression levels for the different MIR156 and MIR157 genes to support for example the selection of MIR156A/C. And explain why mutants for mir157c/d and mir157a/b/c were generated.

**Thanks for this suggestion. We have now provided a graph in Supp. Fig. 1A of the temporal expression patterns at the shoot apex of all detectable *MIR156* and *MIR157* encoded precursor RNAs, as requested. These data are based on our published RNAseq data (Cerise et al, 2023). This independent analysis supports statements in the original version of the manuscript that *MIR156A* and *MIR156C* are the most highly expressed *MIR156* precursors at the apex, and that *MIR157C* and *MIR157D* expression persists later into the inflorescence apex. We have now mentioned this analysis in the Results on line 189 to 196 and 197 to 202 (in the marked-up version). As suggested, we also use this analysis to justify the mutant combinations we used for the miRNA sequencing experiments (line 204-223). The new Supp Fig. 1A also illustrates an interesting temporal pattern of expression of *MIR156D* in apices, suggesting that this gene would also be interesting to analyze genetically. Unfortunately, we do not have mutant alleles for *MIR156D*, and mention this point on Line 195-196.**

- For better readability of the graphs, it would be nice to indicate when the I1 and I2 phases start (in Figures 1 and 2).

**We understand that knowing the approximate onset of the I1 and I2 phases in the analysis shown in Figure 1 would help with interpreting the level of each miRNA at each developmental stage. However, the onset of these stages can only be assessed microscopically by following the morphology of the meristem and the primordia of individual plants as the apex progresses through the floral transition, whereas the data shown in Figure 1 require bulk harvesting of many apices. However, based on confocal imaging experiments previously performed on wild-type plants growing under the same LD conditions and those shown later in the manuscript under SDs, we can estimate the developmental stage of the Col-0 plants at each time point in Figure 1A and 1B. We have now included that information in the legend to Figure 1. Figure 2A and 2B describe the morphological traits of rosette leaves, which are formed exclusively during the vegetative phase. Figure 2C to 2F (now 2D to 2F) show the effect of different mutations on cauline leaf formation, which occurs only during the I1 phase. We have edited the text in the Results section on line 234 and line 263 (of the marked-up version) to make the stages when these leaves are formed clearer.**

- In several of the line graphs, it is very difficult to see what is significant and what not. In Figure 3, the authors even refer to the Suppl. data for this, while it these are relevant data. If they change the graphs to bar graphs in some cases, it will be much easier to indicate significance by using letters.

**We thank the reviewers for this suggestion. We have replaced the line graphs in panels C and D of Figure 3 with bar graphs, and added the statistical significance directly to the graphs to facilitate the interpretation of these results. Supplementary Table S3 has been removed and the text and Figure legend changed accordingly**.

- Figure 2: Why was the mir157a/b/c mutant not included here?

**The mir157a/b/c mutant was analyzed and the corresponding data are presented in the Supplementary Figure S2 and Supplementary Table S2 (line 27 in the summary page). However, the phenotypes were not as strong as for *mir157c mir157d*, for example in cauline leaf number, and mir157a/b/c showed no obvious effects on phyllotaxy or rosette leaf shape (Supplementary Figure S2). Since mir157c mir157d greatly reduces miR157 levels during floral transition and inflorescence development, we emphasized the double mutant in the main figures. The phenotypic data in Supplementary Table S2 further support that *miR157cd* showed the stronger effect on later leaf traits such as cauline leaf number. Showing the triple mutant miR157abc in the supplementary material allowed us to use more space in the main figures for the combinations with stronger phenotypes, and avoided overcomplicating the figures. However, we do mention the leaf trait phenotypes of mir157a/b/c in the Results on line 251 and 266-267.**

- Figure 2C: there is no letter c to indicate significance.

**Thank you, we corrected the mistake.**

- There is a rather abrupt introduction of FUL/SPL15 in the text (lines 250-259) relating to the cauline leave number phenotype. The involvement of FUL here makes it rather complex, and there is no good explanation, nor is it further investigated (e.g. by testing the expression of FUL). The conclusion lines that follow could also ignoring the FUL/SPL15 experiment. It is probably better to delete the experiment on FUL(/SPL15) here (or to work it out better).

**Thank you, we edited this section as suggested and deleted the text on the FUL interaction from the text (lines 255-259 in the original) and from Figure 2D. We retained the text SPL15 on lines 250-255 (in the original; 280-283 in the marked-up version) because that seemed well justified as a target of miR156/miR157 on line 250/314.**

- Lines 289 – 291. ‘The expression of the floral meristem identity gene….., and FUL is activated by SPLs.’ The reasoning of this sentence is not very clear (i.e. there is no logical connection between the first and second part of the sentence).

**We deleted this sentence and rephrased the point (lines 317-322 in the marked-up version) to convey the necessary information.**

- Line 316: ‘similar time to’ should be ‘similar time as’.

**We think that grammatically “similar” should take “to” rather than “as”, but we are happy to follow the PLoS style manual for this.**

- Lines 302 – 305 and 308-310: it seems that FUL and SPL15 are introduced here, while they have already been introduced in lines 250-259. According to our suggestion, you could leave out lines 250-259. However, if these are retained, the text should be adjusted so to timely introduce FUL and SPL15.

**Thanks for pointing this out. As mentioned above, we have deleted the text on FUL that was on lines 255–259, and have remodeled the sentences originally on lines 289-291 describing the relationship between SPLs and *FUL* (now lines 317-322 in the marked-up version). These edited sentences provide an Introduction to SPLs and FUL. Therefore, we think the text from line 302 to 305 (now 332 to 337) mentioning FUL should now be appropriately introduced. We retained the sentence originally on lines 308–310 (now 338-340 in the marked-up version), because this specifically introduces the link between SPL15 and *FUL*. We think now the text should be less repetitive and the information should seem introduced in a timely way.**

- Please comment on how the reporter lines in the different mutant backgrounds were generated. Were the constructs transformed in the different mutant backgrounds and thus all representing independent construct inserts in the genome? In that case, it is important to mention how many independent lines per construct were assessed.

**The marker gene constructs were introduced into the mutant backgrounds by crossing, so the same transgenic event is compared in the Col-0 and different mutant backgrounds. We have made that clearer by adding more information regarding the generation of the transgenic lines described in the manuscript to the Material and Methods section “Plant material and growth conditions”. So, the following lines were generated by crossing the previously published marker lines with the new CRISPR alleles generated in this work: *mir157c-3 mir157d-2 pFUL::9AV:FUL ful-2* and *mir157c-3 mir157d-2 pSPL15::9AV:SPL15*. In Col-0 background, several independent lines were scored for *MIR156B::VNG, MIR156C::VNG, MIR157A::VNG, MIR157C::VNG, MIR157D::VNG* and *AaMIR157C::2HA-mScarlet*. Confocal images of a second independent line for each construct is now included in the Supplementary Figures 4-7.**

- Please also indicate in one of the pictures where Rib zone, PZ and CZ can be found.

**We incorporated this into Figure 4A as suggested.**

**Reviewer #2**

The MIR157–SPL15 module regulates flowering and inflorescence development in Arabidopsis thaliana under short days and in Arabis alpina (PGENETICS-D-25-00166)
The manuscript by Roggen et al. examines the molecular mechanisms underlying the roles of the miR157-SPL15 in flowering regulation under short days in both Arabidopsis thaliana and Arabis alpina. Although it is well-known that the miR156-SPL module functions during vegetative phase changing and flowering time, the role of miR157 in these developmental contexts is less clear. In general, the manuscript is well written and the data are clear and the experiments well planned and executed. However, I have a few questions that may improve the manuscript:

**We thank the reviewer for the positive comments on our work.**

1- In previous publication (He et al., 2018 PloS Genetics), it was shown that miR157 is more abundant than miR156 but has a smaller effect on SPL gene expression than miR156. This may be attributable to the inefficiency with which miR157 is loaded onto AGO1, as well as to the presence of an extra nucleotide at the 5' end of miR157 that is mis-paired in the miR157:SPL duplex. Although the authors showed that miR157 transcripts persisted longer within RISCs late in development (inflorescence development), they did not test the effect of the extra nucleotide in the efficiency of the miRNA:mRNA duplex formation.
Thus, it is difficult to assess whether miR157 effectively regulates SPLs in inflorescence development. Unless I missed it, the manuscript does not provide clear data demonstrating this regulation. If such data are available, it would be helpful to highlight them more explicitly; otherwise, additional experiments could strengthen this conclusion.

**The reviewer raises an important mechanistic point on the potential efficiency difference between miR156 and miR157 in targeting *SPL* mRNAs independently of their temporal and spatial patterns of expression. We had mentioned the difference in loading efficiency into AGO1 of miR156 and miR157 in the Discussion of the original version (line 508-510), but had not mentioned the effect of the extra nucleotide in miR157. The extra nucleotide in miR157c actually extends the complementarity with *SPL15* mRNA by one nucleotide as shown by He et al. (2018). We have now included this information in the new version on lines 629 to 632 (in the marked-up text). We also mention there our indirect evidence that miR157 regulates SPLs based on the early bolting and flowering phenotypes of the *mir157cd* mutants that are expected for plants with increased SPL activity, the genetic evidence that these phenotypes can be partially suppressed by mutations in the *SPL15* and *SPL4* genes, and the confocal microscopy showing that VENUS9A:SPL15 is increased in abundance in *mir157c* mutants (lines 628 to 629). We hope that this explicit description of the indirect evidence that miR157 targets the SPLs and the inclusion of the information on the extra nucleotide answers this point. In the course of these revisions, there was not the time to add an additional experiment.**

2- Neither miR156 nor miR157 mutations seem to affect rosette leaf number in 4wSD plants when comparing with Col-0 and rSPL15 (Fig. 2B). Is there a possible explanation for these phenotypes? The authors showed that the developmental regulation of MIR157D does not follow the general age-associated reduction shown by MIR156 genes (lines 533-534), which suggests that miR157 function is more prominent in adult plants. However, miR157 does accumulate at higher levels in seedlings (He et al., 2018 PloS Genetics). Thus, while miR157 does not affect leaf shape (Supp. Fig. S2), it may have an effect on the plastochron of juvenile plants. Did the authors check that?

**Although visual inspection of the 4wSD plants shown in Figure 2B suggests that there are no major differences in rosette leaf number at this stage, counting the leaves identified differences in rosette leaf number. As the reviewer correctly points out, the miR156/SPL module was previously implicated in plastochron regulation so a difference in rosette leaf number might be expected. Therefore, we now provide a measure of plastochron length in days by dividing the rosette leaf number of the 4wSD plants by the time from germination in days (Figure 2C; lines 253-257). We found that *mir156ac* mutants had a longer plastochron, as expected for plants showing increased SPL activity or reduced miRNA activity (e.g. Wang et al, 2008). By contrast, *mir157cd* mutants did not form leaves more slowly during these 4 weeks and even appeared to form leaves more quickly. These results are consistent with miR156 acting during vegetative development to repress SPL activity, and with miR157c and d being more important later in flowering and inflorescence development. At present, we cannot explain the shorter plastochron of *mir157cd* mutants, but it was previously shown that the expression of *MIR156* from heterologous promoters in different spatial domains had different effects on plastochron length (Wang et al, 2008), suggesting that the spatial patterns of expression of *MIR157C* and *D* may also play a role. We mention our data in the Results (lines 250-253 in the marked-up version) and in the Discussion (lines 602-608).**

3- SPL9 has been shown to promote flowering and it is the primary SPL15 paralogue. The authors mentioned that spl15 mir157c mir157d triple mutants still bolted much earlier than spl15 mutants (lines 583-584), which indicates that other SPLs may be at play. Are the mutations in both paralogues (SPL9 and SPL15) sufficient to suppress mir157 mutant flowering phenotypes? Have the authors examined spl9 spl15 mir157 mutants? If not, this would be an informative experiment to determine whether SPL9 and SPL15 together are the primary contributors to floral transition under SD, or if additional SPL genes are involved.

**Thanks for this point. Yes, the reviewer is right that *SPL9* is a candidate for conferring the residual early bolting of the *spl15 mir157c* and *spl15 mir157cd* mutants. In the Discussion (line 586) of the original version, we mentioned that four other *SPL* genes (*SPL9*, *SPL3*, *SPL4* and *SPL5*) have been reported to promote flowering and that each of these is a candidate for the residual early bolting. The reviewer correctly mentions that *SPL9* is the paralogue of *SPL15*, and therefore is a strong candidate. However, during flowering *SPL9* is expressed in floral primordia whereas *SPL15* is expressed in the SAM and stem (see Hyun et al 2016 for *in situ* hybridizations), so to us *SPL9* seemed less likely to be involved in bolting regulation. By contrast, *SPL4* is strongly expressed in the rib region and SAM during flowering (see Torti et al, 2012 for *in situ* hybridizations). Therefore, we decided first to make the triple mutant *spl15 spl4 mir157c*, and we now show the bolting time of this genotype in Figure 3F and mention it in the Results (lines 350 to 356 of the marked-up version). This triple mutant bolts around 20 days later than *spl15 mir157c*, so *SPL4* is likely to be a major contributor to the residual early bolting in *spl15 mir157c*, but it is still clear that *spl15 spl4* bolts later than *spl15 spl4 mir157c* indicating that yet more SPLs must contribute to the phenotype. We have now included the role of *SPL4* in the Discussion (Lines 634 to 638 of the marked-up version) and specifically make the point that *SPL9* is a paralogue of *SPL15* and remains a candidate to be tested.**

**Reviewer #3**

The Manuscript is well written and all data is supported by the figures and tablles. Here are some minor questions that:

1. The LWR of cauline leave as shown in Figure 2 is lower in miR157d-2 but higher in the double mutant of miR157d-2 miR157c-3, how?

**We agree with the reviewer that the reduction in *mir157d-2* LWR but not in *mir157c-3 mir157d-2* double mutant is intriguing. Based on the expression patterns observed for *MIR157D::VNG* and *MIR157C::VNG* in cauline leaves (Suppl. Figure 7), we propose that the spatial differences in expression of these genes - *MIR157C* in the more distal part of the leaf predominantly on the abaxial side and *MIR157D* most strongly at the base of the leaf - are important for determining cauline leaf morphology in our context. We assume that mir157d mutation affects cauline leaf shape, and this is somehow compensated for by the loss of *MIR157C* function in a different spatial pattern, but until we fully understand the spatial distribution of target SPL transcription factors and their roles in cauline development, this will be difficult to explain more thoroughly. We have now mentioned this effect in the Results (lines 274-275 in the marked-up version) and added sentences to the discussion to highlight this point and the possible explanation mentioned above (lines 659-664 in the marked-up version).**

2. In figure 5A, why no real-time PCR was done on miR156 in long days?

**We have now added in Supplementary Figure S3 the expression levels of *MIR156A* and *MIR156C* in apices in long days. We also now mention it in the Results section line 395 to 396 (in the marked-up version).**
